# Supplementary material for: BInD: Bond and Interaction‐Generating Diffusion Model for Multi‐Objective Structure‐Based Drug Design
Source: Adv Sci (Weinh). 2025 Jul 11;12(35):e02702. doi: 10.1002/advs.202502702 (PMC12463045; doi:10.1002/advs.202502702)
Supplement: Supplementary file 1 — Supporting Information [file ADVS-12-e02702-s001.pdf]

ADVANCED SCIENCE
Open Access

Supporting Information

for *Adv. Sci.*, DOI 10.1002/advs.202502702

BInD: Bond and Interaction-Generating Diffusion Model for Multi-Objective Structure-Based Drug Design

*Joongwon Lee, Wonho Zhung, Jisu Seo and Woo Youn Kim**

Supporting Information

List of Figures

S1. Examples of problematic molecules generated from previous deep SBDD models 3
S2. More examples of generated molecules from BInD 4
S3. Distributions of all atom pairwise distances 6
S4. Distributions of CC bond distances 7
S5. Distributions of angles between CC bonds 8
S6. Distributions of the number of rotatable bonds 9
S7. Strain energy and the number of rotatable bonds 10
S8. Knowledge-based guidance and dynamic interaction network 14
S9. The number of ligand atoms and corresponding pocket volumes 19
S10. Noise schedule for atom type and position, bond type, and NCI type 20

List of Tables

S1. Success rate of different SBDD models on the multi-objective criteria 5
S2. FCD, functional group MAE, and JSD measured on the test set 11
S3. Existence ratios of functional groups and their MAE values on the training set 12
S4. Statistical frequencies of functional groups and their JSD values on the training set 12
S5. Existence ratios of functional groups and their MAE values on the test set 13
S6. Statistical frequencies of functional groups and their JSD values on the test set 13
S7. Ablation study on local geometry guidance 14
S8. Ablation study on interaction guidance 15
S9. Ablation study on dynamic interaction network 15
S10. Sampling efficiency for different SBDD models 16
S11. Input node features of BInD 17
S12. Input edge features of BInD 18
S13. Hyper-parameter setting of BInD 21
S14. Threshold values of knowledge-based guidance terms 22
S15. Proportions of molecules that exceed the threshold values of energy differences between mutant and WT EGFR 23

Failure cases of deep SBDD models

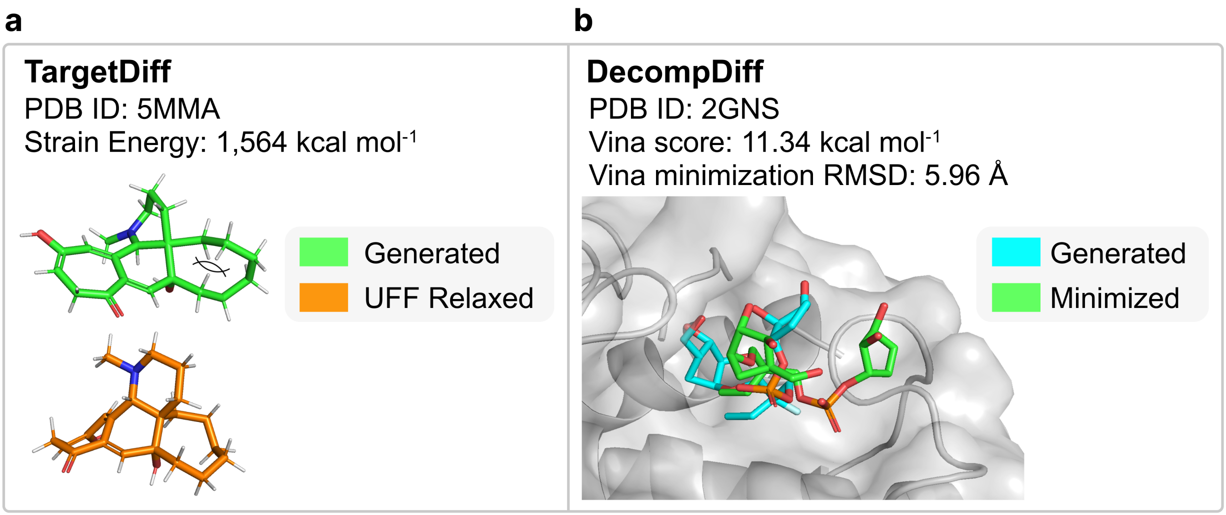

Supporting Figure S1. **Examples of problematic molecules generated from previous deep SBDD models.** **a**, An example of a molecule generated from TargetDiff, where its conformer is sterically strained due to an unreasonable ring structure, compared to the UFF-relaxed conformer. **b**, An example of a molecule generated from DecompDiff, where its binding pose is energetically insecure, leading to a dramatic shift during the energy minimization of its pose.

Examples of generated molecules from BInD

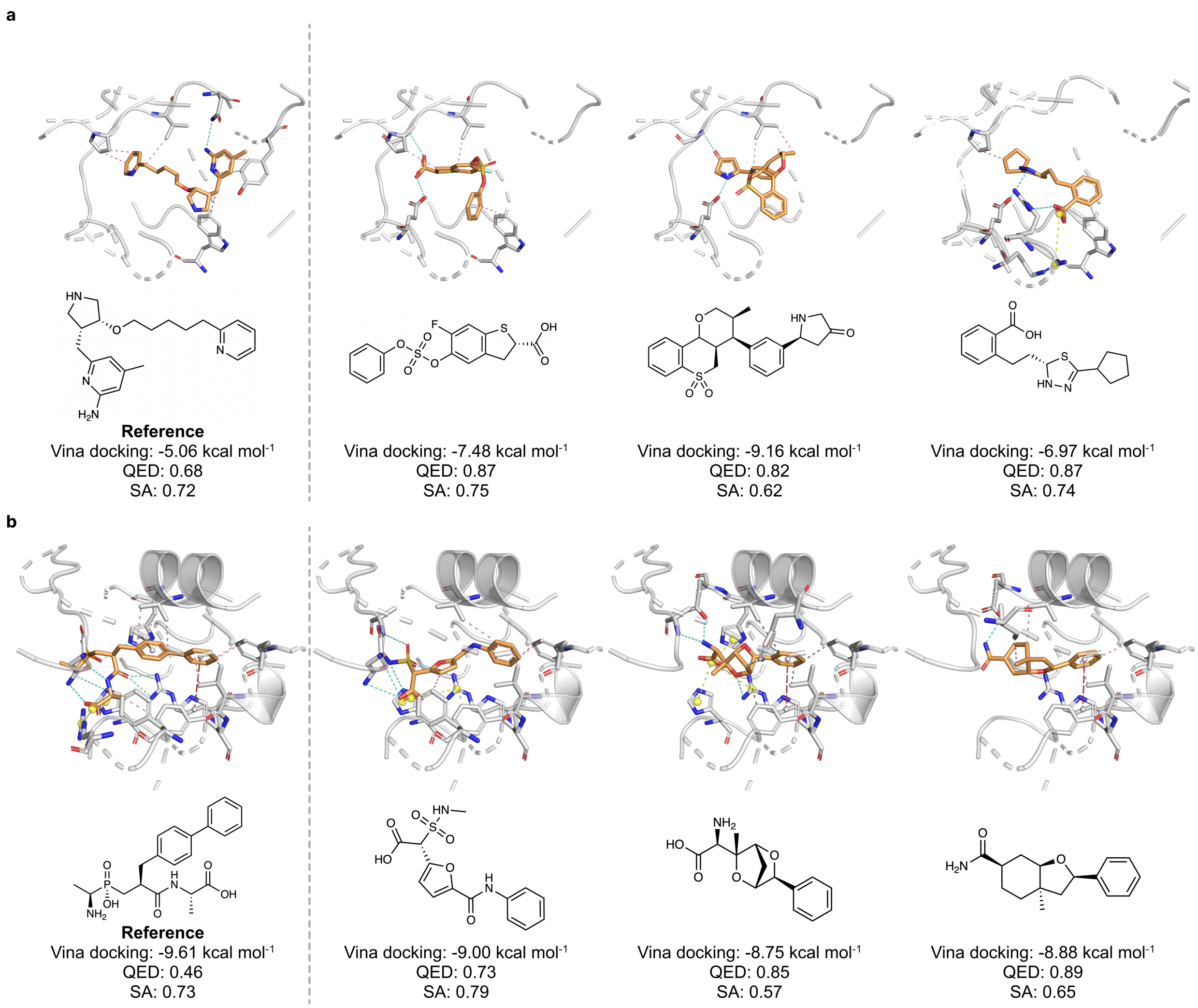

Supporting Figure S2. **More examples of generated molecules from BInD.** Generated 3D structures from **a. 4KCQ** and **b. 1R1H** are shown in orange, with their molecular graphs. Protein pockets are shown in gray, and residues involved in NCIs are depicted together. NCIs, which are analyzed via PLIP software, are depicted as dashed lines, where salt bridges, hydrogen bonds, hydrophobic interactions, and π-π stackings are colored in yellow, light blue, pink, and red, respectively. Vina docking, QED, and SA values are provided, with those of the references.

Additional analysis of generated molecules

Success rates on multi-objective criteria

Criteria	Molecular Property	✓	✓	✓	✓
	Local Geometry	✗	✗	✓	✓
	Interaction	✗	✓	✗	✓
Model	AR	7.5%	5.6%	1.7%	1.3%
	Pocket2Mol	25.6%	4.7%	21.3%	4.0%
	DiffSBDD	8.5%	2.9%	2.9%	1.0%
	TargetDiff	10.9%	5.3%	3.2%	1.5%
	DecompDiff	16.0%	1.8%	5.5%	0.5%
	**BInD**	19.0%	6.7%	12.4%	4.7%

Supporting Table S1. **Success rate of different SBDD models on the multi-objective criteria.** Starting from 10,000 generated molecules, following previous studies [1–3], molecules were filtered using the criteria of QED > 0.25, SA > 0.59, and Vina docking < -8.18 kcal mol^{-1} to satisfy the molecular property criteria. Next, molecules with a difference greater than 1.09 kcal mol^{-1} between the Vina docking and the Vina score were filtered out to assess interaction stability. We set the criterion based on the median difference of the reference molecules. Finally, molecules with strain energy above 200 kcal mol^{-1} were filtered out to ensure favorable local geometry.

Distributions of local geometries

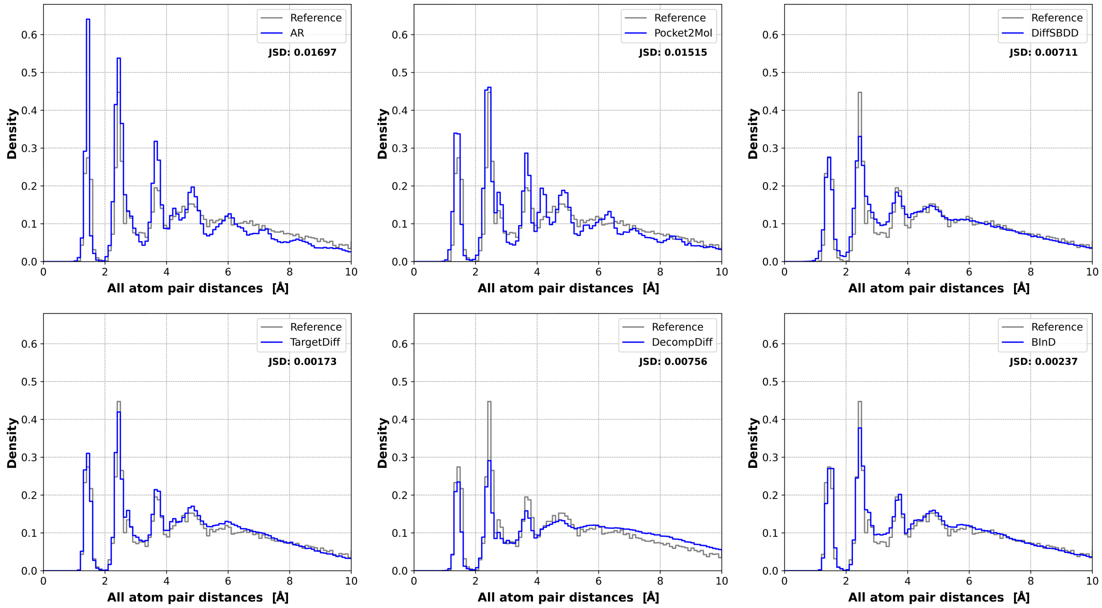

Supporting Figure S3. **Distance distributions of all atom pairs.** JSD values between the reference molecules in the test set are provided.

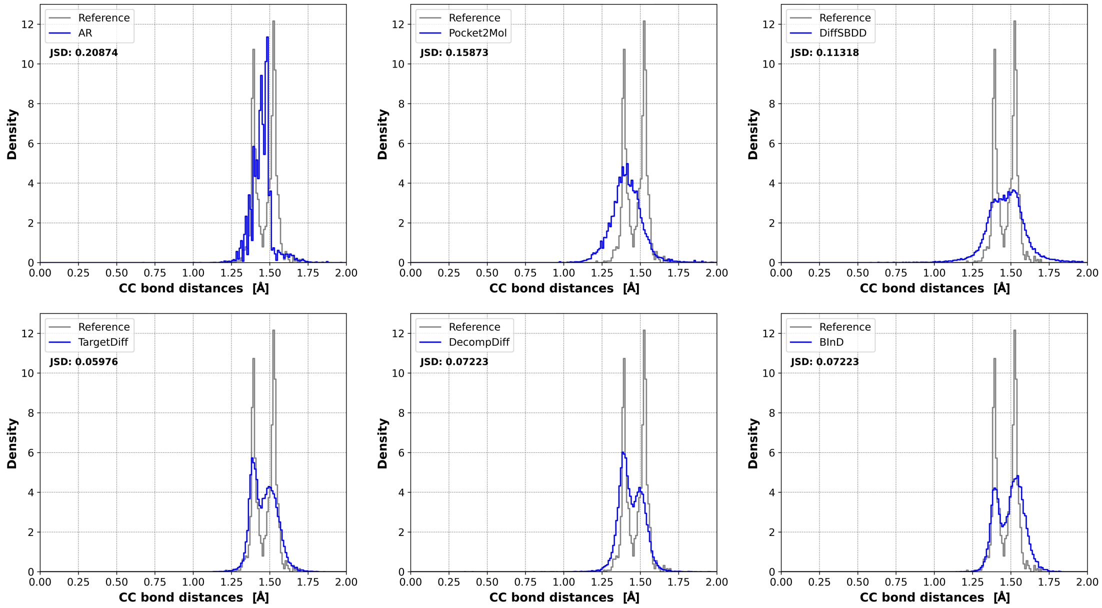

Supporting Figure S4. **Distance distributions of CC bonds.** JSD values between the reference molecules in the test set are provided. BInD represents a clear, doubly peaked pattern with a comparably low JSD value.

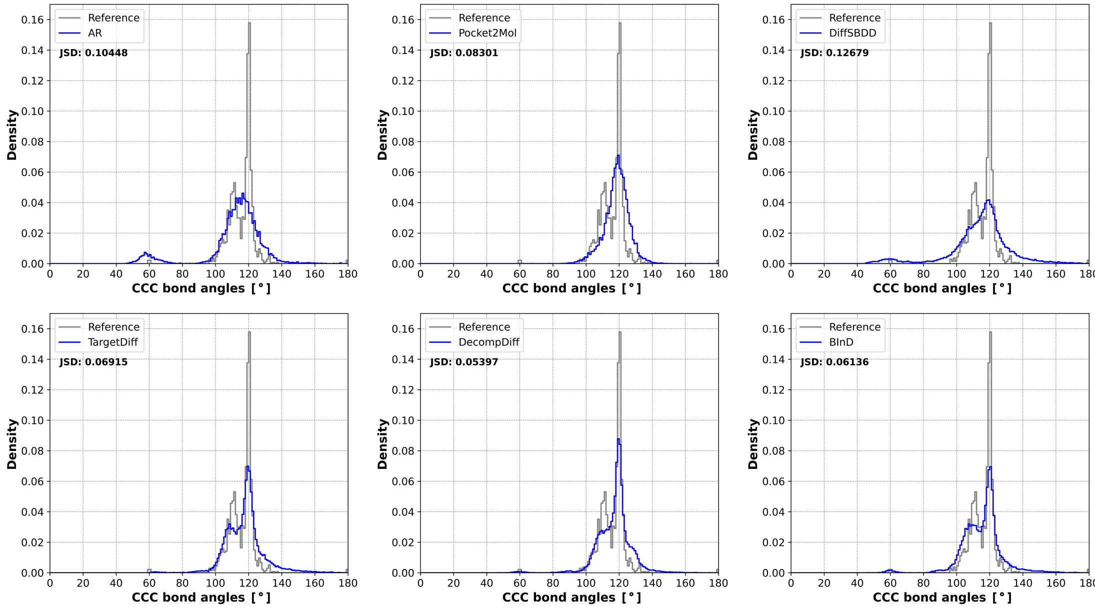

Supporting Figure S5. **Distributions of angles between CC bonds.** JSD values between the reference molecules in the test set are provided.

The number of rotatable bonds and its effect on strain energy

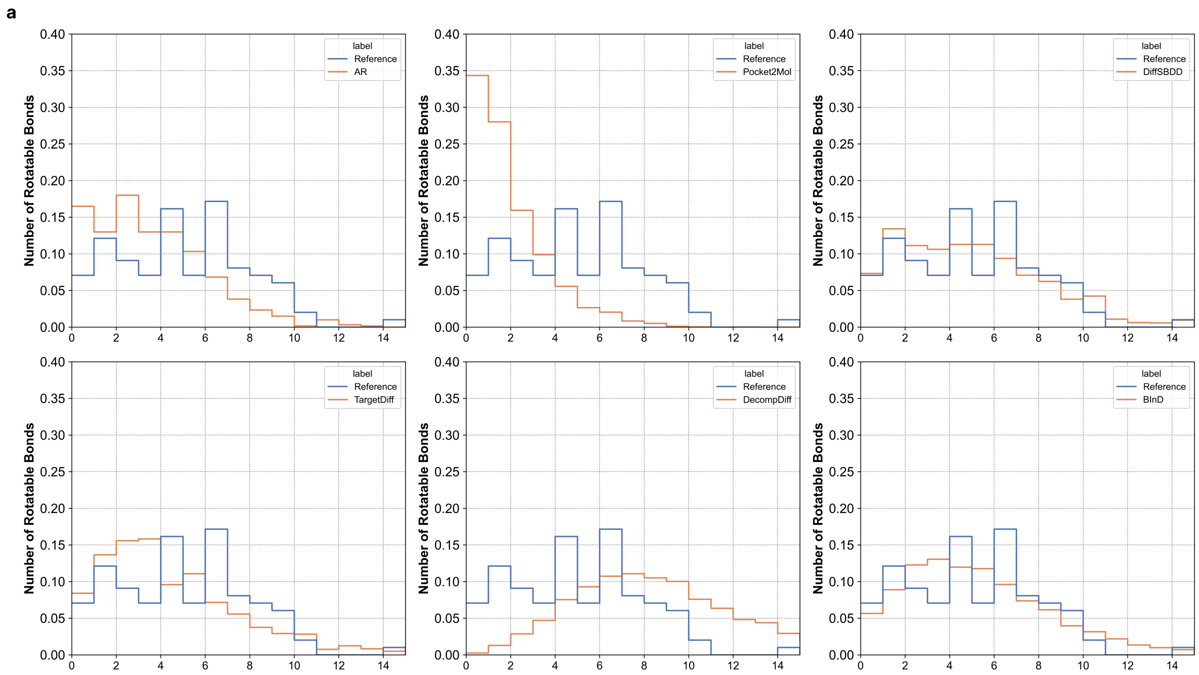

Supporting Figure S6. **Distributions of the number of rotatable bonds from baseline models and BInD.** BInD shows a similar distribution of rotatable bonds while AR and Pocket2Mol generates molecules with less rotatable bonds, and DecompDiff generates molecules with more rotatable bonds than the reference molecules.

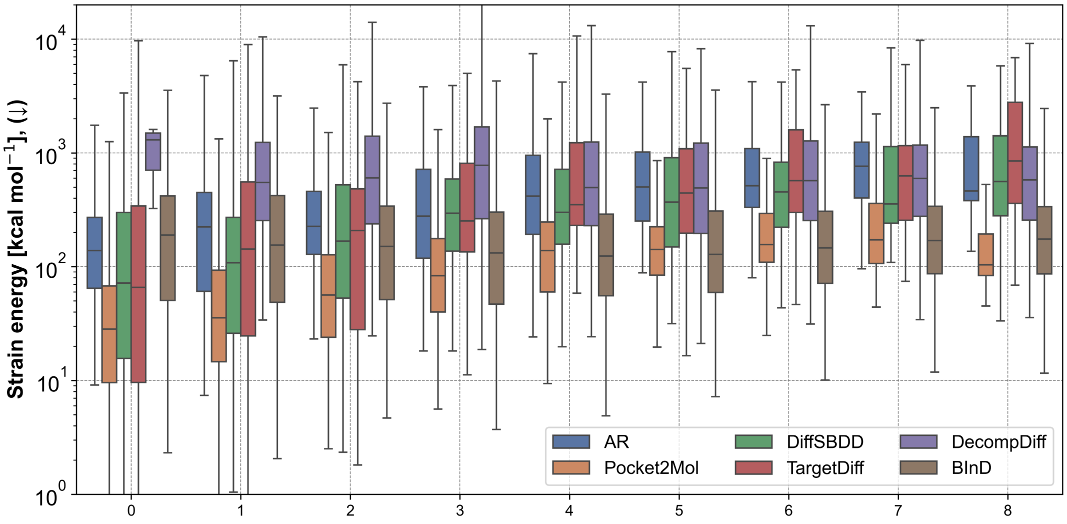

Supporting Figure S7. **Strain energy and the number of rotatable bonds.** Statistics of strain energies are shown as box plots with respect to the different numbers of rotatable bonds for baseline models and BInD. For a small number of rotatable bonds ($n \leq 3$), Pocket2Mol, DiffSBDD, and TargetDiff show lower strain energy than BInD. However, when the number of rotatable bonds is large ($n \geq 4$), their strain energies increase as the number of rotatable bonds increases, implying that large molecular structures with higher complexity may be sterically strained. In contrast, BInD exhibits consistent strain energies regardless of the number of rotatable bonds, competing with Pocket2Mol while outperforming other baseline models when $n \geq 4$. Median values and four-quartile values are indicated with boxes and whiskers.

Molecular properties: FCD and functional group distribution ratio and frequency

Model	FCD (↓)	Func. Group (↓) MAE	Func. Group (↓) JSD
AR	20.08	0.0411	0.2703
Pocket2Mol	22.03	0.0278	0.2950
DiffSBDD	18.68	0.0337	0.2415
TargetDiff	22.51	0.0276	0.2986
DecompDiff	21.12	0.0323	0.2266
**BInD**	**14.80**	**0.0185**	**0.2045**
InterDiff	21.17	0.0468	0.4382
DecompDiff^{ref}	14.54	0.0207	0.1908
**BInD^{ref}**	**12.83**	**0.0175**	**0.1825**

Supporting Table S2. **FCD, functional group MAE, and JSD measured on the test set.** The first six models are reference-free methods and the last three are dependent methods. The best performing models among reference-free methods and reference-dependent models for each benchmark are marked as bold.

Functional Group	Ref.	AR	Pocket2Mol	DiffSBDD	TargetDiff	DecompDiff	**BInD**	InterDiff	DecompDiff ^{ref}	**BInD** $^{\textbf{ref}}$
c1ccccc1	0.7723	0.2196	0.5166	0.2436	0.4079	0.7000	0.5246	0.0498	0.4237	0.4790
NC=O	0.2957	0.1009	0.1052	0.1419	0.2105	0.3825	0.2220	0.1515	0.2151	0.1890
O=CO	0.2564	0.0844	0.2234	0.2438	0.2237	0.2457	0.3010	0.2397	0.2429	0.2586
c1ccncc1	0.0857	0.0412	0.0814	0.0577	0.0395	0.1518	0.0639	0.0054	0.0804	0.0895
c1ncc2nc[nH]c2n1	0.0704	0.0069	0.0014	0.0001	0.0000	0.0007	0.0093	0.0000	0.0114	0.0190
NS(=O)=O	0.0678	0.0000	0.0000	0.0070	0.0000	0.0168	0.0105	0.0000	0.0098	0.0110
O=P(O)(O)O	0.0547	0.0537	0.0069	0.0119	0.0263	0.0261	0.0605	0.0175	0.0408	0.0548
OCO	0.0375	0.0634	0.0169	0.0571	0.0395	0.0496	0.0824	0.1468	0.0616	0.0729
c1cncnc1	0.0356	0.0117	0.0228	0.0063	0.0197	0.0236	0.0161	0.0020	0.0157	0.0362
c1cn[nH]c1	0.0308	0.0157	0.0066	0.0068	0.0000	0.0307	0.0107	0.0000	0.0120	0.0043
O=P(O)O	0.0338	0.0032	0.0000	0.0282	0.0263	0.0236	0.0215	0.0263	0.0380	0.0362
c1ccc2ccccc2c1	0.0318	0.0161	0.0631	0.0046	0.0000	0.0146	0.0237	0.0000	0.0065	0.0133
c1ccsc1	0.0256	0.0000	0.0066	0.0107	0.0000	0.0407	0.0200	0.0000	0.0294	0.0162
N=CN	0.0309	0.0299	0.0124	0.0124	0.0395	0.0171	0.0071	0.0444	0.0106	0.0110
NC(N)=O	0.0189	0.0040	0.0059	0.0113	0.0066	0.0200	0.0098	0.0101	0.0137	0.0105
O=c1cc[nH]c(=O)[nH]1	0.0281	0.0000	0.0069	0.0001	0.0000	0.0000	0.0000	0.0000	0.0000	0.0005
c1ccc2ncccc2c1	0.0142	0.0028	0.0207	0.0031	0.0132	0.0064	0.0076	0.0000	0.0047	0.0100
c1cscn1	0.0210	0.0000	0.0017	0.0074	0.0000	0.0132	0.0080	0.0000	0.0078	0.0076
c1ccc2[nH]cnc2c1	0.0171	0.0004	0.0055	0.0017	0.0000	0.0054	0.0027	0.0000	0.0029	0.0005
c1c[nH]cn1	0.0186	0.0016	0.0138	0.0063	0.0000	0.0254	0.0098	0.0020	0.0124	0.0095
O=[N+][O-]	0.0157	0.0000	0.0038	0.0052	0.0000	0.0011	0.0000	0.0000	0.0012	0.0000
O=CNO	0.0172	0.0028	0.0069	0.0093	0.0132	0.0136	0.0044	0.0081	0.0106	0.0076
NC(=O)O	0.0167	0.0028	0.0093	0.0168	0.0132	0.0136	0.0122	0.0189	0.0092	0.0138
O=S=O	0.0147	0.0000	0.0000	0.0055	0.0000	0.0054	0.0041	0.0000	0.0014	0.0038
c1ccc2[nH]ccc2c1	0.0125	0.0028	0.0031	0.0011	0.0000	0.0057	0.0049	0.0000	0.0041	0.0024
**MAE (↓)**	-	0.0564	0.0383	0.0465	0.0386	0.0227	0.0311	0.0620	0.0329	0.0302

Supporting Table S3. **Existence ratios of each functional group with MAE values compared with the reference molecules in the training set.** 25 most frequent functional groups in CrossDocked2020 are analyzed on their existence probability within a molecule.

Functional Group	Ref.	AR	Pocket2Mol	DiffSBDD	TargetDiff	DecompDiff	**BInD**	InterDiff	DecompDiff ^{ref}	**BInD** $^{\textbf{ref}}$
c1ccccc1	0.3817	0.3307	0.4528	0.2707	0.3780	0.3818	0.3652	0.0690	0.3347	0.3530
NC=O	0.1461	0.1520	0.0922	0.1577	0.1951	0.2086	0.1545	0.2097	0.1699	0.1393
O=CO	0.1267	0.1271	0.1959	0.2709	0.2073	0.1340	0.2095	0.3318	0.1918	0.1905
c1ccncc1	0.0423	0.0620	0.0713	0.0641	0.0366	0.0828	0.0445	0.0075	0.0635	0.0660
c1ncc2nc[nH]c2n1	0.0348	0.0103	0.0012	0.0001	0.0000	0.0004	0.0065	0.0000	0.0090	0.0140
NS(=O)=O	0.0335	0.0000	0.0000	0.0078	0.0000	0.0092	0.0073	0.0000	0.0077	0.0081
O=P(O)(O)O	0.0270	0.0809	0.0060	0.0132	0.0244	0.0142	0.0421	0.0242	0.0322	0.0404
OCO	0.0185	0.0954	0.0148	0.0635	0.0366	0.0271	0.0574	0.2032	0.0487	0.0537
c1cncnc1	0.0176	0.0176	0.0200	0.0070	0.0183	0.0129	0.0112	0.0028	0.0124	0.0267
c1cn[nH]c1	0.0152	0.0237	0.0057	0.0075	0.0000	0.0168	0.0075	0.0000	0.0095	0.0032
O=P(O)O	0.0167	0.0049	0.0000	0.0313	0.0244	0.0129	0.0149	0.0363	0.0300	0.0267
c1ccc2ccccc2c1	0.0157	0.0243	0.0553	0.0052	0.0000	0.0080	0.0165	0.0000	0.0052	0.0098
c1ccsc1	0.0127	0.0000	0.0057	0.0118	0.0000	0.0222	0.0139	0.0000	0.0232	0.0119
N=CN	0.0153	0.0450	0.0109	0.0138	0.0366	0.0094	0.0049	0.0615	0.0084	0.0081
NC(N)=O	0.0093	0.0061	0.0051	0.0125	0.0061	0.0109	0.0068	0.0140	0.0108	0.0077
O=c1cc[nH]c(=O)[nH]1	0.0139	0.0000	0.0060	0.0001	0.0000	0.0000	0.0000	0.0000	0.0000	0.0004
c1ccc2ncccc2c1	0.0070	0.0043	0.0181	0.0034	0.0122	0.0035	0.0053	0.0000	0.0037	0.0074
c1cscn1	0.0104	0.0000	0.0015	0.0083	0.0000	0.0072	0.0056	0.0000	0.0061	0.0056
c1ccc2[nH]cnc2c1	0.0084	0.0006	0.0048	0.0019	0.0000	0.0029	0.0019	0.0000	0.0023	0.0004
c1c[nH]cn1	0.0092	0.0024	0.0121	0.0070	0.0000	0.0138	0.0068	0.0028	0.0098	0.0070
O=[N+][O-]	0.0078	0.0000	0.0033	0.0058	0.0000	0.0006	0.0000	0.0000	0.0010	0.0000
O=CNO	0.0085	0.0043	0.0060	0.0103	0.0122	0.0074	0.0031	0.0112	0.0084	0.0056
NC(=O)O	0.0082	0.0043	0.0082	0.0187	0.0122	0.0074	0.0085	0.0261	0.0073	0.0102
O=S=O	0.0073	0.0000	0.0000	0.0061	0.0000	0.0029	0.0029	0.0000	0.0011	0.0028
c1ccc2[nH]ccc2c1	0.0062	0.0043	0.0027	0.0012	0.0000	0.0031	0.0034	0.0000	0.0032	0.0018
**JSD (↓)**	-	0.2586	0.2387	0.2363	0.2692	0.1821	0.1853	0.4626	0.1766	0.1774

Supporting Table S4. **Statistical frequency of occurrence for each functional group with JSD values compared to the reference molecules in the train set.** 25 most frequent functional groups in CrossDocked2020 are analyzed on their occurrence frequencies within a set of molecules.

Functional Group	Ref.	AR	Pocket2Mol	DiffSBDD	TargetDiff	DecompDiff	**BInD**	InterDiff	DecompDiff^{ref}	**BInD^{ref}**
c1ccccc1	0.5100	0.2196	0.5166	0.2436	0.4079	0.7000	0.5246	0.0498	0.4237	0.4790
NC=O	0.2400	0.1009	0.1052	0.1419	0.2105	0.3825	0.2220	0.1515	0.2151	0.1890
O=CO	0.2000	0.0844	0.2234	0.2438	0.2237	0.2457	0.3010	0.2397	0.2429	0.2586
c1ccncc1	0.1100	0.0412	0.0814	0.0577	0.0395	0.1518	0.0639	0.0054	0.0804	0.0895
c1ncc2nc[nH]c2n1	0.0900	0.0069	0.0014	0.0001	0.0000	0.0007	0.0093	0.0000	0.0114	0.0190
NS(=O)=O	0.0500	0.0000	0.0000	0.0070	0.0000	0.0168	0.0105	0.0000	0.0098	0.0110
O=P(O)(O)O	0.0600	0.0537	0.0069	0.0119	0.0263	0.0261	0.0605	0.0175	0.0408	0.0548
OCO	0.0900	0.0634	0.0169	0.0571	0.0395	0.0496	0.0824	0.1468	0.0616	0.0729
c1cncnc1	0.0400	0.0117	0.0228	0.0063	0.0197	0.0236	0.0161	0.0020	0.0157	0.0362
c1cn[nH]c1	0.0100	0.0157	0.0066	0.0068	0.0000	0.0307	0.0107	0.0000	0.0120	0.0043
O=P(O)O	0.0300	0.0032	0.0000	0.0282	0.0263	0.0236	0.0215	0.0263	0.0380	0.0362
c1ccc2ccccc2c1	0.0200	0.0161	0.0631	0.0046	0.0000	0.0146	0.0237	0.0000	0.0065	0.0133
c1ccsc1	0.0200	0.0000	0.0066	0.0107	0.0000	0.0407	0.0200	0.0000	0.0294	0.0162
N=CN	0.0000	0.0299	0.0124	0.0124	0.0395	0.0171	0.0071	0.0444	0.0106	0.0110
NC(N)=O	0.0200	0.0040	0.0059	0.0113	0.0066	0.0200	0.0098	0.0101	0.0137	0.0105
O=c1cc[nH]c(=O)[nH]1	0.0100	0.0000	0.0069	0.0001	0.0000	0.0000	0.0000	0.0000	0.0000	0.0005
c1ccc2ncccc2c1	0.0000	0.0028	0.0207	0.0031	0.0132	0.0064	0.0076	0.0000	0.0047	0.0100
c1cscn1	0.0200	0.0000	0.0017	0.0074	0.0000	0.0132	0.0080	0.0000	0.0078	0.0076
c1ccc2[nH]cnc2c1	0.0100	0.0004	0.0055	0.0017	0.0000	0.0054	0.0027	0.0000	0.0029	0.0005
c1c[nH]cn1	0.0100	0.0016	0.0138	0.0063	0.0000	0.0254	0.0098	0.0020	0.0124	0.0095
O=[N+][O-]	0.0300	0.0000	0.0038	0.0052	0.0000	0.0011	0.0000	0.0000	0.0012	0.0000
O=CNO	0.0100	0.0028	0.0069	0.0093	0.0132	0.0136	0.0044	0.0081	0.0106	0.0076
NC(=O)O	0.0300	0.0028	0.0093	0.0168	0.0132	0.0136	0.0122	0.0189	0.0092	0.0138
O=S=O	0.0000	0.0000	0.0000	0.0055	0.0000	0.0054	0.0041	0.0000	0.0014	0.0038
c1ccc2[nH]ccc2c1	0.0000	0.0028	0.0031	0.0011	0.0000	0.0057	0.0049	0.0000	0.0041	0.0024
**MAE (↓)**	-	0.0411	0.0278	0.0337	0.0276	0.0323	0.0185	0.0468	0.0207	0.0175

Supporting Table S5. **Existence ratios of each functional group with MAE values compared to the reference molecules in the test set.** The 25 most frequent functional groups in CrossDocked2020 are analyzed on their existence probability within a molecule.

Functional Group	Ref.	AR	Pocket2Mol	DiffSBDD	TargetDiff	DecompDiff	**BInD**	InterDiff	DecompDiff^{ref}	**BInD^{ref}**
c1ccccc1	0.3168	0.3307	0.4528	0.2707	0.3780	0.3818	0.3652	0.0690	0.3347	0.3530
NC=O	0.1491	0.1520	0.0922	0.1577	0.1951	0.2086	0.1545	0.2097	0.1699	0.1393
O=CO	0.1242	0.1271	0.1959	0.2709	0.2073	0.1340	0.2095	0.3318	0.1918	0.1905
c1ccncc1	0.0683	0.0620	0.0713	0.0641	0.0366	0.0828	0.0445	0.0075	0.0635	0.0660
c1ncc2nc[nH]c2n1	0.0559	0.0103	0.0012	0.0001	0.0000	0.0004	0.0065	0.0000	0.0090	0.0140
NS(=O)=O	0.0311	0.0000	0.0000	0.0078	0.0000	0.0092	0.0073	0.0000	0.0077	0.0081
O=P(O)(O)O	0.0373	0.0809	0.0060	0.0132	0.0244	0.0142	0.0421	0.0242	0.0322	0.0404
OCO	0.0559	0.0954	0.0148	0.0635	0.0366	0.0271	0.0574	0.2032	0.0487	0.0537
c1cncnc1	0.0248	0.0176	0.0200	0.0070	0.0183	0.0129	0.0112	0.0028	0.0124	0.0267
c1cn[nH]c1	0.0062	0.0237	0.0057	0.0075	0.0000	0.0168	0.0075	0.0000	0.0095	0.0032
O=P(O)O	0.0186	0.0049	0.0000	0.0313	0.0244	0.0129	0.0149	0.0363	0.0300	0.0267
c1ccc2ccccc2c1	0.0124	0.0243	0.0553	0.0052	0.0000	0.0080	0.0165	0.0000	0.0052	0.0098
c1ccsc1	0.0124	0.0000	0.0057	0.0118	0.0000	0.0222	0.0139	0.0000	0.0232	0.0119
N=CN	0.0000	0.0450	0.0109	0.0138	0.0366	0.0094	0.0049	0.0615	0.0084	0.0081
NC(N)=O	0.0124	0.0061	0.0051	0.0125	0.0061	0.0109	0.0068	0.0140	0.0108	0.0077
O=c1cc[nH]c(=O)[nH]1	0.0062	0.0000	0.0060	0.0001	0.0000	0.0000	0.0000	0.0000	0.0000	0.0004
c1ccc2ncccc2c1	0.0000	0.0043	0.0181	0.0034	0.0122	0.0035	0.0053	0.0000	0.0037	0.0074
c1cscn1	0.0124	0.0000	0.0015	0.0083	0.0000	0.0072	0.0056	0.0000	0.0061	0.0056
c1ccc2[nH]cnc2c1	0.0062	0.0006	0.0048	0.0019	0.0000	0.0029	0.0019	0.0000	0.0023	0.0004
c1c[nH]cn1	0.0062	0.0024	0.0121	0.0070	0.0000	0.0138	0.0068	0.0028	0.0098	0.0070
O=[N+][O-]	0.0186	0.0000	0.0033	0.0058	0.0000	0.0006	0.0000	0.0000	0.0010	0.0000
O=CNO	0.0062	0.0043	0.0060	0.0103	0.0122	0.0074	0.0031	0.0112	0.0084	0.0056
NC(=O)O	0.0186	0.0043	0.0082	0.0187	0.0122	0.0074	0.0085	0.0261	0.0073	0.0102
O=S=O	0.0000	0.0000	0.0000	0.0061	0.0000	0.0029	0.0029	0.0000	0.0011	0.0028
c1ccc2[nH]ccc2c1	0.0000	0.0043	0.0027	0.0012	0.0000	0.0031	0.0034	0.0000	0.0032	0.0018
**JSD (↓)**	-	0.2703	0.2950	0.2415	0.2986	0.2266	0.2045	0.4382	0.1908	0.1825

Supporting Table S6. **Statistical frequency of occurrence for each functional group with JSD values compared with the reference molecules in the test set.** The 25 most frequent functional groups in CrossDocked2020 are analyzed on their occurrence frequencies within a set of molecules.

Ablation studies

To clarify the contribution of our key strategies illustrated in Figure S8 – knowledge-based guidance and dynamic interaction network – to BInD's performance, we conduct ablation studies for each strategy.

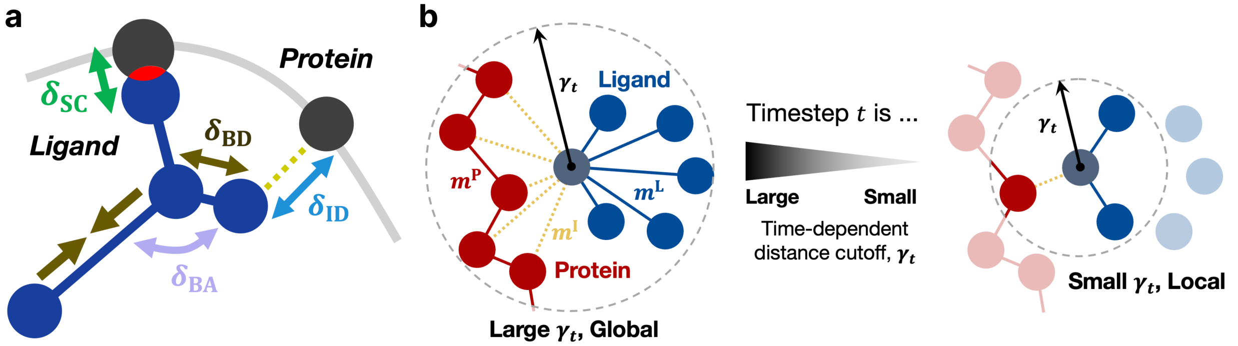

Supporting Figure S8. **Knowledge-based guidance and dynamic interaction network. a**. Simplified illustration of four types of knowledge-based guidance terms: δ_{BD}, δ_{BA}, δ_{ID}, and δ_{SC}. **b**. Illustration of a dynamic interaction network where the distance cutoff for message passing is changing with time, following Equation (26) of the main text. Note that the scheduling of distance cutoff varies for inter- and intra-molecular message passing.

Knowledge-base guidance

Firstly, we ablated the knowledge-based guidance terms to examine each effect. As illustrated in Figure S8, we devised four terms. Among them, bond distance and angle terms can be grouped as ligand local geometry guidance. Meanwhile, the interaction distance and steric clash guidance terms can be grouped as protein-ligand interaction guidance.

Table S7 demonstrates the effect of local geometry guidance on the strain energy of generated molecules. The bond distance guidance term dramatically reduces the strain energy by approximately 11 kcal mol^{-1}. The effect of the bond angle guidance term is minor when the bond distance guidance term is already present; however, the use of both the bond distance and angle guidance yields the best performance in the ligand local geometry. Table S8 shows the effect of interaction guidance on the Vina score and steric clash metrics. Using both the interaction distance and steric clash guidance yields the best result.

**Guidance**		**Strain Energy [kcal mol^{-1}]**, (↓)	
BD	BA	Avg.	Med.
✓	✓	146.7	157.5
✗	✓	162.4	173.4
✓	✗	145.8	161.7
✗	✗	165.5	175.6

Supporting Table S7. **Ablation study on local geometry guidance.** Bond distance (BD) and bond angle (BA) guidance terms are ablated to demonstrate the effect on local geometry. The strain energy was measured through PoseCheck.

Guidance		Vina Score [kcal mol^{-1}], (↓)		Steric Clash, (↓)
ID	SC	Avg.	Med.	
✓	✓	-5.64	-6.22	8.92
✗	✓	-5.44	-5.86	9.10
✓	✗	-5.45	-5.87	8.98
✗	✗	-5.42	-5.94	9.19

Supporting Table S8. **Ablation study on interaction guidance.** Interaction distance (ID) and steric clash (SC) guidance terms are ablated to demonstrate the effect on interaction metrics.

Dynamic interaction network

Next, to ablate the effect of dynamic scheduling of edge radial cutoff distances, we additionally trained two variants of BInD with fixed cutoff values. As described in the Methods section of the main text and Table S13, we employed two pairs of scheduling parameters γ_{min} and γ_{max}, one for ligand edges and the other for interaction edges. We fixed the cutoffs to the minimum values, named BInD^{min}, or the maximum values, named BInD^{max}, while training and sampling.

Table S9 shows the result. When we compare BInD with BInD^{min}, QED and SA scores of BInD^{min} are marginally higher than BInD, but underperformed BInD in Vina scores and strain energies. The in-place Vina score of BInD^{min} is exceptionally high, which is about 0.5 kcal mol^{-1} higher than that of BInD. When comparing BInD with BInD^{max}, Vina scores of BInD^{max} are slightly better than BInD, but fall behind BInD in QED, SA scores, and strain energies. In particular, the strain energy of BInD^{max} was significantly high, about 2.2 times higher than that of BInD. The results align well with the philosophy of dynamic distance cutoff: large cutoff values are advantageous for considering global semantics, while small cutoff values help the model focus on refinement. Thus, scheduling the radius cutoffs from large values to smaller values through timesteps is beneficial in balancing global interaction and local geometry, resulting in the highest success rate.

Model	Ligand edge		Interaction edge		Vina Score		Vina Min.		Vina Dock		QED		SA		Strain Energy		Success Rate
	γ_{min}	γ_{max}	γ_{min}	γ_{max}	Avg.	Med.	Avg.	Med.	Avg.	Med.	Avg.	Med.	Avg.	Med.	Avg.	Med.	
BInD^{min}	4.0	4.0	7.0	7.0	<u>-5.14</u>	<u>-5.89</u>	-6.54	-6.67	-7.66	-7.66	0.53	0.54	0.67	0.68	178.5	194.9	3.8%
BInD^{max}	8.0	8.0	10.0	10.0	-5.82	-6.44	-6.75	-6.81	-7.56	-7.60	0.49	0.49	0.62	0.61	<u>324.1</u>	<u>347.4</u>	3.6%
**BInD**	4.0	8.0	7.0	10.0	-5.64	-6.22	-6.56	-6.80	-7.46	-7.66	0.50	0.54	0.65	0.66	146.7	157.5	4.7%

Supporting Table S9. **Ablation study on dynamic interaction network.** We used fixed radius cutoff values [Å] as the minimum values (BInD^{min}) or the maximum values (BInD^{max}) to isolate the effect of dynamic scheduling. The benchmark scores that significantly underperform BInD are underlined.

Sampling efficiency

We benchmarked the duration to generate 100 molecules for each test target of BInD with Pocket2Mol and TargetDiff using a single RTX A4000 GPU. As shown in Table S10, BInD's inference is faster than Pocket2Mol and TargetDiff by a factor of 1.6 and 1.7, respectively.

Model	**Speed [s mol^{-1}]**,(↓)	
	Avg.	Med.
Pocket2Mol	21.57	22.33
TargetDiff	22.58	23.33
**BInD**	13.45	13.62

Supporting Table S10. **Sampling efficiency for different SBDD models.** We generate 100 molecules for 10 randomly selected protein pockets from the test dataset and measured the average and median duration required to generate 100 molecules for each protein. All sampling was performed using a single RTX A4000 GPU.

Input features

Node features

The input node features of ligand and protein atoms, h_i^{L} and h_j^{P}, are used as described in the Table S11. Each feature is represented as a one-hot vector of a corresponding category. Only an atom type is used for a ligand atom feature, while more informative features are used for a protein atom feature by concatenating every one-hot vector. The resulting dimensions of ligand and protein atom features are 10 and 40, respectively.

**Ligand atom feature**, h_i^{L}	Available list
Atom type	C, N, O, F, P, S, Cl, Br, I, *absorbing* (one-hot)

**Protein atom feature**, h_j^{P}	Available list
Atom type	C, N, O, S, *else* (one-hot)
Atom num H	0, 1, 2, 3, 4, 5, *else* (one-hot)
Formal charge	-2, -1, 0, 1, 2 (one-hot)
Amino acid type	G, A, V, L, I, C, M, F, Y, W, P, S, T,<br>Q, N, D, E, H, R, K, *else* (one-hot)
Is C_α?	0 or 1

Supporting Table S11. **Input node features of BInD.** The node features of ligand and protein atoms and their available item lists were used to construct one-hot vectors. *absorbing* indicates an absorbing type and *else* is used when no item list matches the data type.

Edge features

The input intra and inter-edge features, e_{ij}^{L}, e_{ij}^{P}, and $\mathbf{i}_{ij}$, are used as described in the Table S12. Each feature is represented as a one-hot vector of a corresponding category. For intra-edges of protein and ligand graphs, chemical bond types are used as features. NCI types are used as features for inter-edges between a protein and ligands. Note that the subject of directed NCI is set to protein; for instance, hydrogen bond donor type implies the donor is at the protein atom side. The resulting dimensions of intra and inter-edge features are 5 and 7, respectively.

**Bond (intra-edge) feature**, e_{ij}^{L} and e_{ij}^{P}	Available list
Bond type	Single,<br>Double,<br>Triple,<br>Aromatic,<br>*absorbing* (one-hot)
**NCI (inter-edge) feature**, $\mathbf{i}_{ij}$	Available list
NCI type	Salt bridge anion,<br>Salt bridge cation,<br>Hydrogen bond donor,<br>Hydrogen bond acceptor,<br>Hydrophobic interaction,<br>π-π stacking,<br>*absorbing* (one-hot)

Supporting Table S12. **Input edge features of BInD** The edge features of ligand and protein bonds (intra-edge) and NCIs (inter-edge), along with their available item lists, are used to construct one-hot vectors. *absorbing* indicates an absorbing type and *else* is used when no item list matches the data type.

Prior distribution of the number of ligand atoms

Diffusion-based molecular generative models require a predefined number of atoms, while the size of the molecule in a protein pocket relies on the size of the protein pocket. While previous diffusion-based SBDD models [1, 4] utilized the farthest distance of protein atoms to estimate pocket size, these methods rely on a reference ligand, which is usually defined as residues within 10 Å. To achieve a reference ligand-free estimation of pocket volume, we used POcket Volume MEasurer 2 (POVME2) [5]. POVME2 analyzes the volume of a protein pocket by fitting a voxelized grid into the pocket. We ran POVME2 on the full training dataset with the default settings to obtain pocket volume estimates. Subsequently, we estimated the distribution of POVME2 to the number of atoms by applying a Gaussian to each data point. The Gaussian smearing standard deviation was set to 100 and 3 for a pocket volume and the number of heavy atoms, respectively. During generation, we first measure a pocket volume and then obtain the given volume's marginal distribution. Within the distribution, we randomly sample the number of atoms. Figure S9. depicts the distribution of the pocket volume and number of heavy atoms along with the test set marked.

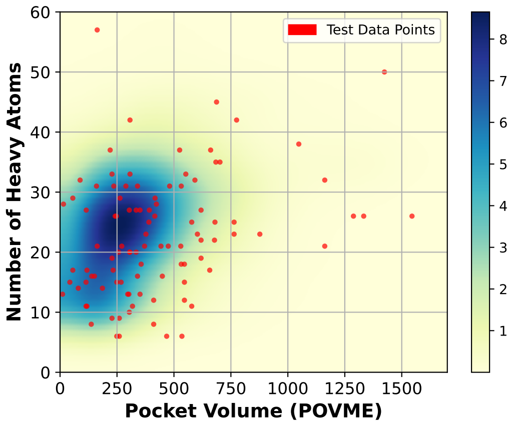

Supporting Figure S9. **The number of ligand atoms and corresponding pocket volumes.** Distribution of the number of ligand heavy atoms and corresponding pocket volumes measured by POVME software. The red dots indicate the test data points.

Training and Sampling Details

Noise schedule

Following MolDiff [6], we use the modified sigmoid noise schedule, which is in the form of:

$$s = \frac{(s_T - s_1)}{\text{sigmoid}(-w) - \text{sigmoid}(w)}, \tag{1}$$

$$b = 0.5 \times (s_1 + s_T - s), \tag{2}$$

$$\bar{\alpha}_t = s \times \text{sigmoid}\left(-w\left(\frac{2t}{T} - 1\right)\right) + b, \tag{3}$$

where s_1, s_T, and w are hyper-parameters for different features. Intuitively, NCIs still strongly depend on the distance between atoms, but their distribution is less peaked than bond lengths. Consequently, a noise schedule in the order of bond type, NCI type, and finally, atom type and position. We choose the hyper-parameters as described in Table S13. and Figure S10., which shows the signal-to-noise schedule (SNR) change with respect to the diffusion timestep.

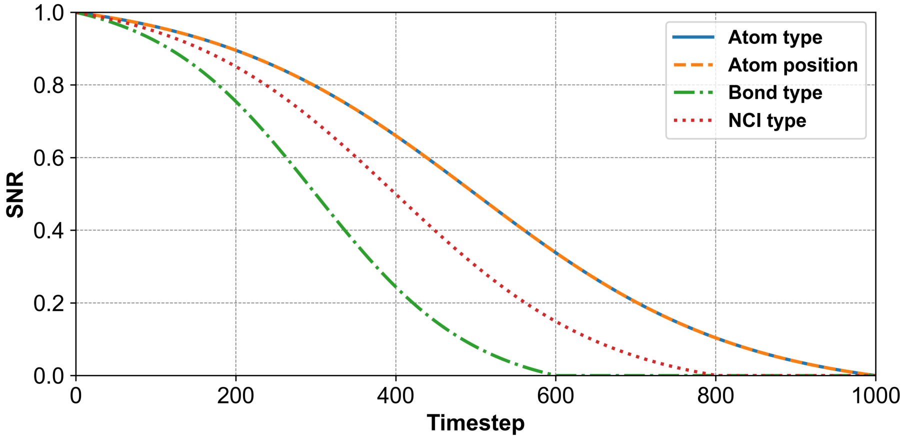

Supporting Figure S10. **Noise schedule for atom type and position, bond type, and NCI type.** The signal-to-noise ratio ($\bar{\alpha}_t$) decreases as timestep increases, following the order of bond type $\rightarrow$ NCI type $\rightarrow$ both atom type and positions.

Architecture details

BInD comprises six layers of $E(3)$-equivariant dynamic interaction networks with node and edge updates. For each layer of message passing, node processing, and edge processing layers, we used two MLP layers with layer normalization. For stable training, we used a hyperbolic tangent activation function. A single neural network at each layer embeds the distance between each node and concatenates onto the message. Two MLP layers did the final prediction with layer normalization.

During training, we scaled the loss for atom, bond, and NCI types to 100 to ensure consistent loss magnitudes. The model was optimized using the Adam optimizer [7]. Evaluation in the validation set was conducted every five epochs of training, and if no improvement was observed over 10 evaluations, the learning rate was reduced by a factor of 0.8. Training was performed on a single NVIDIA A100 GPU, requiring approximately 60 hours for convergence.

	Description	Value
Diffusion	Number of diffusion timestep	1000
	Atom type and position noise schedule	$(w, s_1, s_T) = (3, 0.9999, 0.0001)$ for $t \in [0, 1000]$
	Bond type noise schedule	$(w, s_1, s_T) = (3, 0.9999, 0.001)$ for $t \in [0, 600]$
	NCI type noise schedule	$(w, s_1, s_T) = (3, 0.9999, 0.001)$ for $t \in [0, 800]$
Model	Embedding dimension of node features	96
	Embedding dimension of edge features	96
	Message dimension	128
	Timestep embedding dimension	32
	Number of DIN layers	6
	γ for protein edge	6.0
	$\gamma_{\min}$ and $\gamma_{\max}$ for ligand edge	[4.0, 8.0]
	$\gamma_{\min}$ and $\gamma_{\max}$ for interaction edge	[7.0, 10.0]
Train	Initial learning rate	1e-3
	β_1 and β_2 of Adam optimizer	[0.95, 0.99]
	Weight decay	1e-16
	Batch size	64
	Gradient clipping threshold	8.0
Sampling	Number of resampling	4
	Resampling timestep range	[600, 1000]
	Bond distance guidance, a_{BD}	0.02
	Interaction distance guidance, a_{ID}	0.02
	Bond angle guidance, a_{BA}	0.05
	Steric clash guidance, a_{SC}	0.02

Supporting Table S13. **Hyper-parameter setting of BInD.** Hyper-parameters are categorized by diffusion, model, train, and sampling.

**Bond distance**	$d_{\text{BD}}^{\text{min}}$	$d_{\text{BD}}^{\text{max}}$
	1.2 Å	1.9 Å
**NCI distance**, type κ	$d_{\text{ID},\kappa}^{\text{min}}$	$d_{\text{ID}_\kappa}^{\text{max}}$
salt bridge	2.8 Å	7.5 Å
hydrogen bond	2.4 Å	4.1 Å
hydrophobic interaction	2.0 Å	4.0 Å
π-π stacking	3.0 Å	7.0 Å
**Bond angle**	$d_{\text{BA}}^{\text{min}}$	
	$1.2\sqrt{2}$ Å	
**Steric clash**	$d_{\text{SC}}^{\text{min}}$	
	0.5 Å	

Supporting Table S14. **Threshold values of knowledge-based guidance terms.** Threshold values for each knowledge-based guidance term are provided. Note that $d_{\text{BA}}^{\text{min}}$ is set as a distance when two bonds with a distance of $d_{\text{BD}}^{\text{min}}$ are separated in 90°.

Additional analysis on mutant-selective EGFR inhibitor design

Model	Δ**Energy [kcal mol^{-1}]**			
	> 2	> 3	> 4	> 5
Random	0.33%	0.00%	0.00%	0.00%
Pocket2Mol	0.33%	0.33%	0.00%	0.00%
TargetDiff	3.67%	1.67%	0.00%	0.00%
**BInD**	8.33%	1.00%	0.00%	0.00%
**BInD$^{\mathbf{opt1}}$**	13.67%	3.67%	0.67%	0.33%
**BInD$^{\mathbf{opt2}}$**	20.33%	5.67%	1.67%	0.67%

Supporting Table S15. **Proportions of molecules that exceed the threshold values of energy differences between mutant and WT EGFR.** Among 300 molecules generated from each model or randomly gathered from the training set, the binding energy differences are evaluated between mutant and WT EGFR, $\Delta E = E_{\mathrm{WT}} - E_{\mathrm{mutant}}$, with Vina docking. We then calculated the proportions of molecules that possess a greater difference than each of the threshold values. A higher proportion indicates a higher number of mutant-selective molecules.

Supporting References

[1] Jiaqi Guan et al. "DecompDiff: Diffusion Models with Decomposed Priors for Structure-Based Drug Design". In: *International Conference on Machine Learning*. PMLR. 2023, pp. 11827–11846.

[2] Yutong Xie et al. "Mars: Markov molecular sampling for multi-objective drug discovery". In: *arXiv preprint arXiv:2103.10432* (2021).

[3] Siyu Long et al. "Zero-shot 3d drug design by sketching and generating". In: *Advances in Neural Information Processing Systems* 35 (2022), pp. 23894–23907.

[4] Jiaqi Guan et al. "3D Equivariant Diffusion for Target-Aware Molecule Generation and Affinity Prediction". In: *International Conference on Learning Representations*. 2023.

[5] Jacob D Durrant et al. "POVME 2.0: an enhanced tool for determining pocket shape and volume characteristics". In: *Journal of chemical theory and computation* 10.11 (2014), pp. 5047–5056.

[6] Xingang Peng et al. "MolDiff: Addressing the Atom-Bond Inconsistency Problem in 3D Molecule Diffusion Generation". In: *Proceedings of the 40th International Conference on Machine Learning*. Ed. by Andreas Krause et al. Vol. 202. Proceedings of Machine Learning Research. PMLR, 23–29 Jul 2023, pp. 27611–27629. URL: `https://proceedings.mlr.press/v202/peng23b.html`.

[7] Diederik P Kingma and Jimmy Ba. "Adam: A method for stochastic optimization". In: *arXiv preprint arXiv:1412.6980* (2014).
